# Roots of Progress: Uncovering Cerebellar Ataxias Using iPSC Models

**DOI:** 10.3390/biomedicines13092121

**Published:** 2025-08-30

**Authors:** Michela Giacich, Valentina Naef, Filippo Maria Santorelli, Devid Damiani

**Affiliations:** Neurobiology and Molecular Medicine Units, IRCCS Fondazione Stella Maris, 56128 Pisa, Italy; michela.giacich@fsm.unipi.it (M.G.); valentina.naef@fsm.unipi.it (V.N.); filippo3364@gmail.com (F.M.S.)

**Keywords:** iPSCs, cerebellum, cerebellar ataxias, neuronal differentiation, disease modeling

## Abstract

The inaccessibility of human cerebellar tissue and the complexity of its development have historically hindered the study of cerebellar ataxias, a genetically diverse group of neurodegenerative disorders. Induced pluripotent stem cell (iPSC) technology offers a powerful solution, enabling the generation of patient-specific cerebellar models that retain individual genetic backgrounds. This review examines recent progress in iPSC-derived cerebellar models and their application in relation to major hereditary ataxias, including Friedreich’s ataxia, ataxia–telangiectasia, and spinocerebellar ataxias (SCAs). These models have provided valuable insights into disease mechanisms and supported the development of therapeutic strategies, such as gene therapy and high-throughput drug screening. However, challenges remain, particularly in achieving the full maturation of cerebellar cell types and incorporating microglial interactions. Moreover, emerging evidence suggests that neurodevelopmental alterations may act as early contributors to degeneration. Despite the current limitations, the advancement of patient-derived iPSC cerebellar models holds great promise for uncovering novel disease pathways and for driving precision medicine approaches in cerebellar ataxia research.

## 1. Introduction

**Figure 1:**
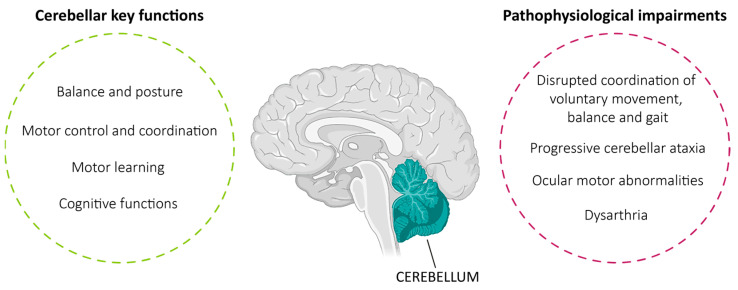
Human cerebellum and its key functions. Schematic representation highlighting the human cerebellum with its physiological functions and common pathological impairments.

The cerebellum is a key component of the central nervous system (CNS), known to be involved in essential tasks such as motor control coordination, balance and regulation of posture [1]. Recent evidence suggests that it is also implicated in higher cognitive processes, expanding our understanding of its role within broader neural networks (Figure 1) [2,3]. As a result, the intricate structure and functions of the human cerebellum, combined with the difficulty of accessing its tissue, have significantly hindered research into cerebellar disorders. The development of the human cerebellum involves a highly orchestrated temporal sequence of events, beginning with the prenatal specification of neural progenitor territories [4]. Initially, the isthmic organizer (IsO) at the midbrain–hindbrain boundary (MHB) sends signals through the fibroblast growth factor (FGF) and wingless/integrated (WNT) signaling pathways, inducing the regional specification and proliferation of progenitor cells. Later, two major germinal zones, the ventricular zone (VZ) and the rhombic lip (RL), give rise to different cerebellar neuronal populations. The VZ mainly produces GABAergic neurons like Purkinje cells (PCs), interneurons and glial cells, while the RL generates glutamatergic neurons like granule cells (GCs), unipolar brush cells, and deep cerebellar nuclei (DCN) projection neurons [4]. These neurons migrate to their respective positions in deeply coordinated molecular patterns driven by master genes like *ATOH1* in the RL and *KIRREL2* for VZ-derived cells [5]. PCs are the most studied, as they are known to both play a crucial role in the integration and refinement of movement signals and be the most susceptible cell type in cerebellar neurodegenerative processes. PCs display extensive and complex dendritic arborizations, establishing synaptic connections with excitatory climbing fibers coming from the inferior olive and receiving inhibitory stimulus from many GC parallel fibers, the most abundant neurons in the cerebellum, in turn obtaining afferences from the brainstem and spinal cord [6]. Overall, the mature cerebellum is connected with the cerebral cortex, spinal cord, and thalamus, allowing it to modulate both motor and cognitive functions. PCs project into the neocortex through DCN and the thalamus, and the neocortex relays back the signal to the cerebellum via pontine nuclei. These pathways were traditionally viewed as closed-loop circuits, but recent studies propose a broader role in coordinating communication between different cortical areas [7,8]. In addition, the cerebellum is thought to enhance synchronization between task-relevant cerebral cortical areas by modulating neuronal oscillations, helping with coherent and context-specific neural interactions [9].

Structural and functional impairments of the cerebellum have been implicated in several disorders [10,11,12,13]. Among them, one of the most common is represented by a group of conditions collectively known as cerebellar ataxias, generally characterized by disrupted coordination of voluntary movement, balance and gait. This wide group of disorders can result from diverse underlying genetic mutations, often leading to shared degenerative processes, significantly impairing patients’ quality of life [14]. The most common inherited ataxia is Friedreich’s ataxia (FRDA), characterized by progressive gait instability, sensory ataxia, dysarthria, eye movement abnormalities and cardiomyopathy [15]. FRDA results in most cases from an autosomal recessive GAA trinucleotide repeat expansion in the first intron of the *FXN* (frataxin) gene, leading to transcriptional silencing [16]. As frataxin protein is essential for mitochondrial function and iron–sulfur (Fe-S) cluster biogenesis, its deficiency causes mitochondrial dysfunction and neurodegeneration, particularly affecting the dorsal root ganglia, spinal cord and cerebellar pathways [17,18,19].

Another major group of autosomal-dominant ataxias is represented by the spinocerebellar ataxias (SCAs), characterized primarily by progressive cerebellar ataxia, dysarthria and ocular motor abnormalities. Among the over 50 forms, each SCA subtype arises from mutations in specific genes, often involving CAG trinucleotide repeat expansions that cause toxic polyglutamine (polyQ) extensions and lead to misfolding and aggregation of the encoded protein [20]. Thus, the most prevalent SCAs, like SCA1, SCA2 and SCA3, share a common molecular etiology due to a pathological repeat expansion in their respective genes—*ATXN1* (ataxin-1), *ATXN2* and *ATXN3*. For instance, SCA1 is defined by transcriptional dysregulation due to the gain-of-function interactions of mutant *ATXN1* with transcriptional regulators such as Capicua (CIC) and splicing factor RBM17, resulting in suppressed expression of genes involved in glutamatergic neurotransmission [21]. SCA2 primarily affects PCs, inducing a large range of molecular abnormalities like calcium homeostasis dysregulation, impaired autophagy, oxidative stress, and synaptic dysfunction. Experimental evidence from SCA2 mouse models showed that PC dysfunctions—characterized by abnormally slow firing and progressive degeneration—lead to broader cerebellar network disruption and motor impairments [22]. SCA3, also known as Machado–Joseph disease (MJD), is the most common dominantly inherited ataxia, accounting for between 15% and 45% of all SCAs worldwide [23]. Together with the typical cerebellar ataxia symptoms, SCA3 is characterized by pyramidal and extrapyramidal alterations, peripheral neuropathy and mild cognitive impairments [24]. Also, in this case, the polyQ expansion was reported to lead to protein misfolding, aggregation of mutant ataxin-3, disruption of the ubiquitin–proteasome system, transcriptional dysregulation and mitochondrial dysfunction in several in vivo and in vitro models [25].

Besides the SCA family, an autosomal recessive form of ataxia is ataxia–telangiectasia (A-T), an early-onset multisystem neurodegenerative disorder characterized by rapidly progressing cerebellar ataxia, oculocutaneous telangiectasias, immunodeficiency and increased cancer susceptibility [26]. A-T is caused by mutations in the *ATM* (ataxia–telangiectasia-mutated) gene that completely inactivate or eliminate the ATM protein [27]. The *ATM* gene encodes a serine–threonine kinase essential for the DNA damage response (DDR), specifically for repairing double-strand breaks (DSBs) [28]. Indeed, in response to DNA damage, ATM is activated and phosphorylates targets as P53, CHK2, H2AX, and others, triggering a signaling cascade that coordinates the cell cycle, DNA repair, apoptosis, and oxidative stress response [29]. Beyond its canonical nuclear functions, ATM also plays roles in mitochondrial redox homeostasis, lysosomal trafficking and autophagy regulation, reflecting its broader influence on neuronal survival and cellular metabolism [30]. A peculiar hallmark of A-T’s pathology consists of the selective and progressive degeneration of PCs and cerebellar granule neurons, whereas other neuronal types remain relatively spared.

Other rarer forms of ataxia are becoming recognized worldwide. Autosomal recessive spastic ataxia of Charlevoix–Saguenay (ARSACS) is a rare neurodegenerative disorder caused by mutations in the *SACS* gene [31]. This gene encodes a large multi-domain cytoplasmic protein known as sacsin. While the exact physiological role of sacsin remains only partially understood, recent studies suggest that it may be a co-chaperone involved in regulating mitochondrial fission/fusion regulation, intermediate filament assembly and protein quality control, thus being linked to mitochondrial function and neuronal cytoskeletal integrity [32]. Indeed, loss of sacsin disrupts the mitochondrial fission/fusion balance, causes neurofilament bundling and impairs exosomal transport, leading to neurodegeneration [33]. At the clinical level, ARSACS manifests as an early-onset progressive cerebellar ataxia, usually associated with spastic paraplegia due to pyramidal tract impairments and peripheral neuropathy. PCs are particularly vulnerable to degeneration, displaying axonal swelling, mitochondrial dysfunction, and cytoskeletal abnormalities [34].

Although genetic causes and molecular defects underlying these forms of ataxia have been identified, there are still major gaps in our understanding of how these pathways affect different CNS networks and influence disease progression, emphasizing the need for further research. Though research in animal models is universally considered essential and valuable, it often fails to fully recapitulate the complex cellular and functional properties unique to human CNS development and related pathologies. For instance, unlike human patients, some mouse models of A-T lack a high incidence of tumors as well as clear morphofunctional impairments [35]. Additionally, finding a reliable model for SCA pathologies posed a significant challenge for researchers of the field [36,37,38,39]. Consequently, developing robust in vitro human models of cerebellar tissue is critical for a deep understanding of these processes to find and screen new therapies. Recent advancements in human induced pluripotent stem cell (hiPSC) technology have opened up new avenues for modeling cerebellar development and diseases in culture and evaluating cellular impairments (e.g., mitochondrial dysfunctions, morphological alterations) that can occur in particular neurological diseases in specific cerebellar populations, such as Purkinje and granule cells [40]. In addition, the advent of tridimensional differentiation now offers the possibility to generate iPSC-derived cerebellar organoids, potentially mimicking key developmental and functional characteristics of the human cerebellum and providing a powerful platform to investigate disease mechanisms, drug responses and therapeutic strategies [41,42,43]. A known limitation of iPSC-derived models is the lack of complete cellular heterogeneity and integrated network functionality occurring within the whole brain. However, iPS cells represent a powerful tool for research, as they can be derived from easily accessible adult tissues. This way, it is possible to preserve the patient’s genetic background, bypassing ethical concerns linked to embryonic stem cells and reducing the need for animal models. Their capacity for indefinite expansion and pluripotent differentiation provides scalable tools suitable for investigating etiopathological mechanisms at the cellular level, enabling the development of drug discovery and personalized medicine [44,45]. Moreover, recent breakthroughs in iPSC-derived 3D culture allow the generation of more complex structures, termed “assembloids”, in which the combination of 3D aggregates with different brain identities may partially overcome the limitation of iPSC-derived models in capturing integrated network activity.

Finally, a crucial cell population generally missing in 2D and 3D iPS-derived models is represented by microglial cells, which are not derived from the ectodermal layer. The latest development of reliable protocols for the generation of these immune cells [46] permits co-cultured models and therefore the achievement of an increased level of complexity, as recently reported in several papers [47,48,49].

These systems can effectively recapitulate the cerebellar complexity, including functional features like electrophysiological activity and long-term maturation that closely mirror in vivo conditions. The present review aims to critically analyze the current literature about iPSC-derived cerebellar models, summarizing existing knowledge on human cerebellar development and specifically focusing on their utility in studying cerebellar ataxias. Thus, this review’s objective is to identify key gaps in our understanding of ataxia pathophysiology and evaluate the strengths and limitations of 2D and 3D iPSC-derived cultures in addressing these challenges in order to inspire future project directions and explore potential implications for the development of innovative therapeutic strategies.

## 2. The Developmental Blueprint of the Cerebellum

**Figure 2:**
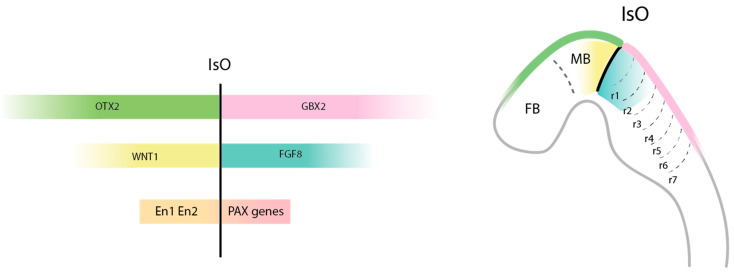
Molecular orchestration of the isthmic organizer. The IsO orchestrates midbrain and anterior hindbrain patterning through the expression of key signaling molecules: *OTX2* is expressed anteriorly in the midbrain (MB), and *GBX2* is expressed posteriorly in the hindbrain. These domains induce the expression of additional factors, such as WNT1 and FGF8 (right panel), and subsequently, *En1/En2* and different *PAX* genes, respectively which further support establishment of the IsO boundary. FB, forebrain; r, rhombomere.

Although the cerebellum has relatively simple histological organization and was traditionally considered solely a motor structure, its essential role in cognitive functions is now widely recognized [50]. Early cerebellar development begins during patterning events in the neural tube, establishing positional information along the anteroposterior and dorsoventral axes, which guides the formation of brain compartments. The midbrain–hindbrain boundary (MHB), also known as the IsO (Figure 2), plays a pivotal role in cerebellar development by orchestrating midbrain and anterior hindbrain patterning, laying the foundation for cerebellar anlagen formation. This region, indeed, emerges thanks to the opposing expression of *OTX2* (orthodenticle homeobox 2) in the anterior neural plate, representing the future forebrain and midbrain, and *GBX2* (gastrulation brain homeobox 2) posteriorly, representing the prospective hindbrain and spinal cord [51,52]. These domains induce the expression of key morphogens, such as FGF8 (fibroblast growth factor 8) and WNT1 (Wingless Int), which are critical for cerebellar primordium formation. *WNT1* is expressed in the caudal midbrain near the *OTX2*-positive domain and cooperates with FGF8 to establish and maintain the IsO. WNT1 regulates the expression of *Engrailed* genes (*EN1* and *EN2*), considered the master genes for specification of the identity of the midbrain and anterior hindbrain regions [53]. Thus, isthmic-derived FGF8 represents a central organizer signal essential for cerebellar induction, promoting both regional identity and cell proliferation. The role of this morphogen is so fundamental that zebrafish mutants lacking Fgf8 completely fail to develop a cerebellum [50]. Moreover, the activation of several *PAX* (paired-box) genes in the MHB in response to FGF8 signaling further supports the establishment of the IsO and cerebellar development [54]. The competence of neural tissue to respond to FGF8 signaling depends on additional regulatory elements, such as *OCT3/4* (octamer binding transcription factor 3/4) and *Canopy1* [50]. As development proceeds, the cerebellar primordium begins to expand laterally, shaping the wing-like morphology characteristic of the future cerebellum. Simultaneously, the adjacent hindbrain separates along the dorsal midline, forming two lateral subdomains and a central layer of non-neuronal epithelial tissue, known as the roof plate, which will later develop into the choroid plexus. The roof plate plays an important morphogenetic and signaling role, expressing the transcription factor LMX1α (LIM homeobox transcription factor 1 alpha) and secreting members of the TGF-β (transforming growth factor beta) superfamily, including GDF7 (growth differentiation factor 7), BMP6 and BMP7 (bone morphogenetic proteins). These morphogens induce the expression of the pro-neural gene *ATOH1* (atonal homolog 1) in two distinct regions: the cerebellar rhombic lip and the hindbrain rhombic lip [55]. At this point, the emergence of two cerebellar-specific germinal zones, the VZ and the RL, gives rise to specific classes of neurons that form the basis for the functional architecture of the mature cerebellum.

### 2.1. Patterning of Cerebellar Neuronal Subtypes

Located at the roof of the fourth ventricle, the VZ begins to express the pro-neural transcription factors ASCL1 (achaete–scute homolog 1) and PTF1α (pancreatic transcription factor 1 alpha), a master regulator of GABAergic neuron specification, defining a cerebellar proliferating domain committed to generating a variety of inhibitory neuronal subtypes, including PCs, the sole cerebellar cortex output neurons, interneurons such as Golgi, basket, stellate and candelabrum cells, and small inhibitory neurons of the DCN [56].

**Figure 3:**
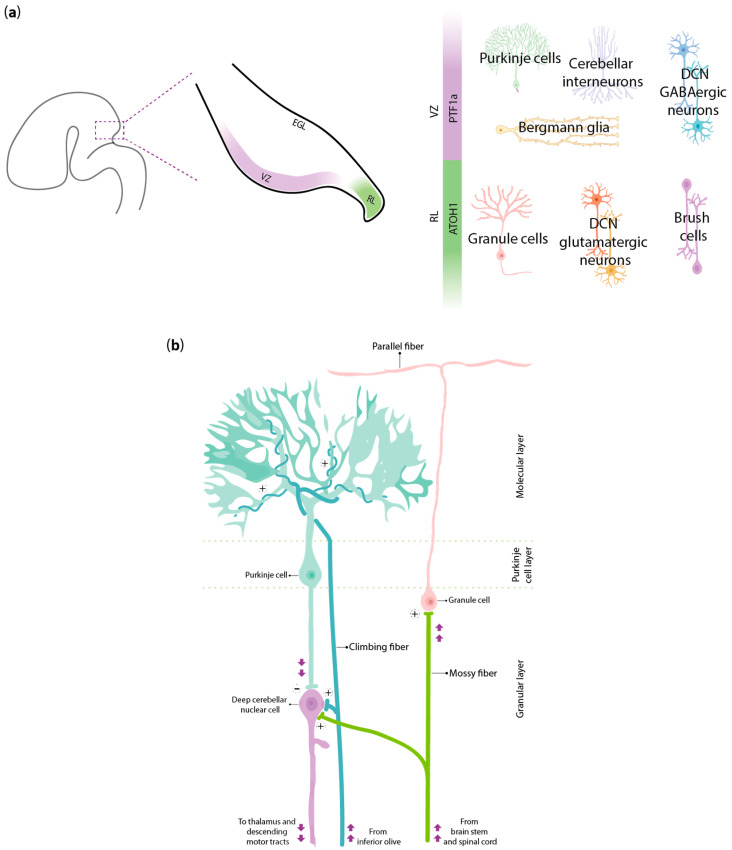
Lineage specification and functional integration of cerebellar neurons during development. (**a**) The distinct germinal zones of the developing cerebellum guide the fate of emerging neuronal populations. PTF1α-expressing VZ gives rise to PCs, cerebellar interneurons, DCN GABAergic neurons and Bergmann glia. ATOH1-expressing RL gives rise to GCs, DCN glutamatergic neurons and unipolar brush cells. (**b**) The cerebellum receives two main classes of afferent input: mossy fibers (green) and climbing fibers (turquoise). Climbing fibers synapse with PCs, while mossy fibers synapse with granule cells within glomeruli and indirectly influence PCs via parallel fibers. PCs represent the sole output of the cerebellar cortex, sending inhibitory projections to the DCN. EGL, external granular layer.

Interestingly, the VZ itself is not homogeneous, as it undergoes further subdivision into rostral and caudal domains, each specifying distinct neuron types based on differential gene expression. The caudal VZ exhibits strong expression of *E-cadherin* and co-expression of *Neurogenin-1*, giving rise to SKOR2+ (SKI family transcriptional corepressor 2) PCs, which are essential for cerebellar output and organization [57]. The rostral VZ lies adjacent to a PAX2+ domain and primarily produces small inhibitory neurons of the DCN. Thus, precise molecular gradients within the VZ tightly regulate the identity of cerebellar GABAergic neuron subtypes. In parallel, the cerebellar RL, located at the caudal edge of the cerebellar primordium, expresses the transcription factor ATOH1, giving rise to glutamatergic excitatory neurons, including GCs and large projection neurons of the DCN [58]. This functional dichotomy within the PTF1α+ VZ and the ATOH1+ RL represents a foundational principle of cerebellar development, establishing the excitation and inhibition balance necessary for the proper function of the mature cerebellum [59] (Figure 3a).

While key aspects of cerebellar cortical development begin prenatally, neuronal migration and maturation continue after birth, contributing to the formation of the layered architecture of the cerebellar cortex. PCs migrate radially from the VZ toward the pial surface, establishing the Purkinje cell layer. Immature excitatory DCN neurons, generated from the ATOH1+ RL, migrate centrally to form the DCN, the main relay stations of cerebellar output. Later, granule cell precursors (GPCs) arise from the RL and migrate tangentially across the cerebellar surface to form the external granule cell layer (EGL). From there, GCPs eventually migrate inward to the internal granule layer (IGL), differentiating into mature GCs [50]. At the molecular level, the process of cerebellar layering is controlled by a group of signaling molecules:

- Semaphorins (Sema4C, Sema4G, Sema6A), directing the radial migration of GCPs, ensuring correct laminar positioning [60,61].
- Reelin, a secreted glycoprotein highly expressed in the EGL, regulating PC alignment and overall cerebellar layering. Its deficiency results in disorganized layering and ataxia-like phenotypes [62].
- Sonic hedgehog (SHH), secreted by PCs, acts as a potent mitogen for GCPs and drives expansion of the EGL that shapes cerebellar foliation [63].

This signaling axis is vital for normal development and can become oncogenic when dysregulated [50].

### 2.2. Integration of Inputs and Outputs of Cerebellar Wiring

The cerebellum receives two principal classes of afferent inputs: mossy fibers and climbing fibers (Figure 3b). Climbing fibers originate exclusively from the inferior olive, a brainstem nucleus located within the medulla [64]. These axons travel contralaterally to the cerebellum, where they synapse with the PC dendrites in a remarkably topographic manner: each inferior olivary subnucleus projects to specific PC stripes within the cerebellar cortex. During early development, individual PCs are innervated by multiple climbing fibers. However, through a postnatal pruning process, all but one climbing fiber are eliminated, resulting in the adult mono-innervation pattern, which is essential for the fidelity of signal transmission in mature cerebellar circuits. It is hypothesized that the axon guidance mechanisms regulating this elegant system involve the activity of the Eph/ephrin signaling pathway [65,66]. Mossy fibers arise from different sources across the brainstem and spinal cord, including the pontine nuclei, reticular formation, spinal cord nuclei and other pre-cerebellar structures. These fibers convey multisensory and contextual information, terminating within the cerebellar granule cell layer and forming complex specialized synaptic hubs known as glomeruli, where mossy fiber terminals interact with the dendrites of both GCs and Golgi interneurons [67]. Mossy fiber inputs are organized into parasagittal domains, which, although less defined than climbing fibers, often align with PC compartments. Interestingly, mossy fibers initially form transient synaptic contacts with PC clusters in the embryonic and neonatal cerebellum. Generation and migration of GCs induce mossy fibers to shift their connectivity, detaching from PCs and establishing stable synapses within the newly formed glomeruli of the IGL. Nonetheless, the initial PC architecture may serve as a guide for afferent map formation. Overall, while climbing fibers act as strong and temporally precise inputs that induce complex spikes in PCs, mossy fibers provide the contextual and sensory landscape required for fine-tuning motor commands and associative learning. The alignment of both afferent systems with the Purkinje cell layer emphasizes a unified morphofunctional framework that highlights cerebellar modular processing and supports its roles in motor coordination, sensorimotor integration and cognitive functions [6,66].

Regarding efferent connections, the only cerebellar cortical output originates from PCs and goes to the DCN. Once activated by converging excitatory inputs, PCs deliver inhibitory signals to the DCN, thereby modulating the excitatory output that the DCN relay to other brain regions. Although PCs are inhibitory, the overall network can promote motor activity: reduced inhibition from PCs increases the firing rate of DCN neurons, enhancing the excitatory output [68]. Each functional region of the cerebellum reaches out to a corresponding nucleus in the DCN, which subsequently interacts with other brain regions. For instance, the dentate nucleus sends projections to the motor thalamic nuclei, which consequently relay signals to the motor and premotor cortices. The resulting circuit forms the cerebello-thalamo-cortical pathway, crucial to modulate planning, execution and cognitive modulation of movement. The interposed nucleus instead sends projections to the red nucleus and motor thalamus, regulating distal limb coordination. The fastigial nucleus receives input from the vermis and sends efferent signals to the vestibular and reticular nuclei, influencing postural control. Finally, DCN neurons project into the inferior olive, completing a feedback loop crucial for learning and timing [69].

The vestibulocerebellum diverges slightly from this organization. Instead of projecting into the DCN, the PCs in this region directly target the vestibular nuclei in the brainstem, allowing for rapid modulation of balance and ocular reflexes [4,56,70].

## 3. iPSCs for Modeling Cerebellar Ataxias

**Figure 4:**
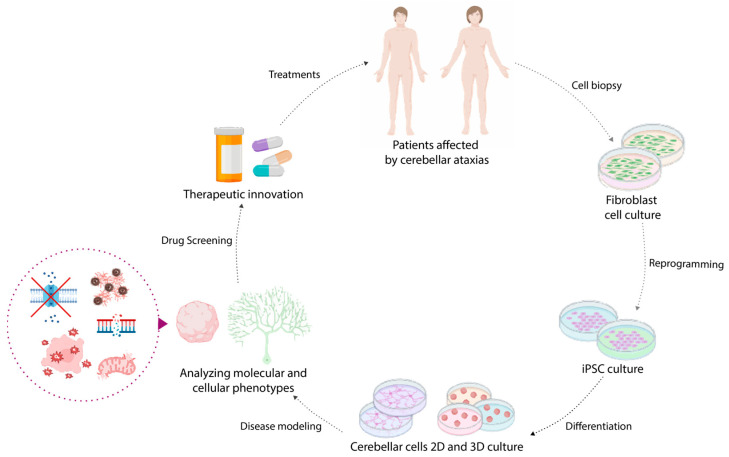
Schematic overview of the iPSC-based disease modeling workflow for cerebellar ataxias. Patient-derived somatic cells are reprogrammed into induced pluripotent stem cells (iPSCs), which can be differentiated into disease-relevant cerebellar cell types. These cells can be used to generate 2D and 3D in vitro models that recapitulate key molecular and cellular phenotypes of cerebellar ataxias, such as mitochondrial dysfunction, DNA repair deficit, ion channel alterations, protein accumulation and oxidative stress. Through these models, disease mechanisms can be investigated and therapeutic compounds screened, contributing to the development of personalized medicine approaches for neurodegenerative disorders.

Studying cerebellar disorders is especially challenging due to the complexity of cerebellar development, its mature structure, and the limited accessibility of this region. iPSC technology offers a powerful tool for modeling these disorders, allowing in-depth investigation of their molecular and cellular mechanisms and promoting the development of a platform to test putative novel therapeutic strategies (Figure 4). Here, we will focus on recent iPSCs models of cerebellar ataxias, a clinically and genetically vast group of neurological disorders primarily affecting motor coordination. Compared to neurodegenerative diseases such as Parkinson’s disease, Alzheimer’s disease and Huntington’s disease, hardly any studies have focused on generating iPSC-based models of cerebellar ataxias. There are many known genetic causes of different ataxias, including the autosomal-dominant and -recessive subtypes, most of them sharing pathogenic pathways like mitochondrial dysfunction, DNA repair deficiencies, and ion channel alterations [71]. These mechanisms often converge on the impairment of PCs, making them a key target for disease modeling.

### 3.1. Advances and Challenges in Human iPSC-Derived Cerebellar Models

The difficulties in producing mature functional cerebellar cells and the challenges of replicating complex 3D architectures and cell–cell interactions, maybe due to the incomplete understanding of cerebellar ontogeny and human-specific developmental processes, highlighted the urgent need for the optimization of in vitro human-specific cerebellar modeling strategies [72]. To the end of studying cerebellar development and for possible use in cell replacement therapy, many protocols were initially based on generating an expandable and transplantable population of Purkinje neuron progenitors derived from mouse embryonic stem cells, capable of maturing in vivo within the adult mouse cerebellum. For instance, one of the first works aimed to generate PCs from mouse embryonic stem cells by mimicking the self-inductive microenvironment of the IsO [73]. Using the surface marker Neph3 for prospective selection, these cells exhibited characteristic morphology, mature marker expression (e.g., GluRδ2), and functional electrophysiological properties akin to in vivo Purkinje neurons. Upon transplantation, they integrated orthotopically into the Purkinje cell layer and established synaptic connectivity [73]. However, these models had some limitations, such as the limited integration of progenitors within the host and, of course, the non-human derivation of these cells [74]. Considering all these issues, researchers introduced a reliable and reproducible protocol for generating PCs from iPSCs, based on the exposure of cells to a defined cocktail of signaling molecules—insulin, FGF2 and TGFβ receptor inhibitors—to mimic the self-inductive signaling of the IsO. This procedure allowed the production of progenitor cells expressing *EN1*, a marker of mid-hindbrain identity, after 3 weeks in culture. Further patterning until day 35 produced distinct cerebellar progenitor domains, characterized by *ATOH1* (RL lineage) and *PTF1α* expression (VZ lineage). A known limitation for these protocols is the lack of efficiency in the final maturation of Purkinje neurons, obtainable only via co-culture with neonatal mouse cerebellar GCs [75]. In a more recent study, a series of optimizations in culture conditions, together with mouse cerebellar astroglia co-culture, enhanced the yield and viability of iPSC-derived PCs, particularly under long-term culture conditions [76]. When cultured under optimized conditions, a subset of differentiated cells exhibited typical Purkinje-like morphology, with extensive dendritic arborization and axonal projections. These cells also expressed Purkinje-specific transcription factors and cytoplasmic markers, indicating successful lineage specification (e.g., *LHX5*, *SKOR2*, *RORA* and *CALB1*). Moreover, co-culturing with glial cells appeared critical for maintaining cellular polarity, promoting neurite outgrowth and reducing cell death. Cells grown on three-dimensional Matrigel-based scaffolds and supported by neurotrophic factors showed improved branching and survival compared to monolayer culture [76].

However, various protocols have been optimized from that of Muguruma et al. [43]. This is a pioneering protocol for generating 3D culture system of cerebellar tissue using human embryonic stem cells (hESCs). It involves sequential treatment of FGF2 and insulin, promoting midbrain–hindbrain fate, and FGF19 and SDF1 addition to induce RL-like and VZ-like domains. These structures self-organize into polarized neuroepithelial rosettes with PCs and GCs, mimicking first-trimester cerebellar ontogeny. Other researchers have developed and applied a self-organization-based 3D culture method to generate cerebellar tissues from human pluripotent stem cells, including both embryonic stem cells and induced pluripotent stem cells. One protocol builds upon the serum-free floating culture of embryoid body-like aggregates with quick reaggregation (SFEBq) that recapitulates early mammalian neural development. The resulting organoids show the layered architecture characteristic of the cerebellar plate and contain markers of both RL- and VZ-derived lineages [77]. Atamian et al. presented an advanced protocol for the generation and long-term maturation of human cerebellar organoids that seems to recapitulate the full cellular diversity of the fetal cerebellum [41]. This protocol points out dual SMAD inhibition and caudalization with the WNT-agonist and FGF8 instead of FGF2, facilitating the generation of both excitatory and inhibitory progenitors. Long-term maintenance and functional maturation (four months longer than Muguruma) allow better replication of human-specific cerebellar traits. Another recent protocol adapts the Muguruma method to create cerebellar organoids, focusing on modeling brain tumors, like medulloblastoma [42]. It combines 3D differentiation with innovative genetic engineering via electroporation, specifically utilizing the PiggyBac transposon system. Moreover, the protocol incorporates lineage tracing by using Cre-loxP recombination systems driven by cell-type-specific promoters (e.g., *SOX2* or *S100B*), allowing for precise tracking of cerebellar progenitor populations. These tools support a wide range of downstream applications, including disease transformation modeling, live imaging, orthotopic transplantation and drug screening.

### 3.2. Friedreich’s Ataxia Disease Modeling Using iPSCs

iPSCs derived from patients with FRDA were differentiated into functional neuronal progenitors, showing normal mitochondrial functions, no enhanced susceptibility to cell death, and effective differentiation capabilities into cerebellar regions of host adult rodent brains upon transplantation [78]. Such models have highlighted the potential for studying disease mechanisms and developing new potential therapies. For example, the GLP-1R (glucagon-like peptide-1 receptor) agonist, an exenatide commonly used to treat type 2 diabetes, has been suggested as a potential therapeutic compound for FRDA [79,80,81]. Exenatide treatment of FRDA-patient-derived neurons led to a consistent and significant increase in the FXN mRNA and protein levels, suggesting that GLP-1R activation can upregulate frataxin expression. Mechanistically, the increase in FXN is linked to the induction of cAMP and NRF2 (NF-E2-related factor), which are known to mediate cellular stress responses and mitochondrial processes, modulating oxidative stress [82,83]. Moreover, treatment with exenatide restored the mitochondrial membrane potential, improved ATP production and reduced oxidative stress in FRDA sensory neurons, as measured by MitoTracker and reactive oxygen species (ROS) assays [84].

### 3.3. Genetic Heterogeneity and iPSC-Based Models of Spinocerebellar Ataxias (SCAs)

SCAs are among the most genetically heterogeneous disorders, including over 40 subtypes with mutations in genes encoding ion channels, transcription factors and other proteins (e.g., ATNX1, ATXN2, ATXN3, CACNA1A, CACNA1G), leading to polyglutamine expansions. While animal models have shed light on disease mechanisms, their limitations in mimicking human cerebellar architecture and disease progression have led to the rise of patient-derived iPS-based models for the generation of cerebellar neuron subtypes like PCs, which are selectively vulnerable in SCAs. Thus, researchers focused on modeling several SCA subtypes, including SCA1, SCA2, SCA3 and SCA6, using patient-derived iPSCs, identifying disease-relevant phenotypes, such as impaired dendritic arborization and reduced viability in PCs, transcriptional dysregulation of calcium channel components, bioenergetic failure due to mitochondrial dysfunction, and altered glutamate-induced calcium signaling leading to proteolytic cleavage and aggregation of mutant proteins [85,86].

In later years, iPSCs have been derived from different SCA2 patient fibroblast lines. SCA2 results in progressive cerebellar atrophy and degeneration of pontine and spinal motor neurons due to pathological polyQ expansions in *ATXN2*, which disrupts calcium signaling, leading to excitotoxicity and impaired long-term depression (LTD), essential for cerebellar learning [87]. PolyQ accumulation has actually been observed to disrupt calcium channels’ functions, like inositol 1,4,5-trisphosphate receptors (IP3R1), ryanodine receptors and voltage-gated calcium channels [88]. iPSCs derived from SCA2 patients have been instrumental in uncovering disease mechanisms and testing therapeutic strategies, as these models closely recapitulate patient-specific neuronal phenotypes, allowing exploration of early molecular events and intervention points [89]. iPSC-derived neural stem cells (NSCs) from SCA2 patients showed atypical clumping into cyst-like structures instead of neural rosettes, and they also exhibited a propensity for spontaneous differentiation during the early stages of culture. Moreover, longitudinal imaging revealed that SCA2 neurons had significantly reduced survival compared to controls. A reduction of *ATXN2* expression already occurs in SCA2 NSCs, consistent with previous findings in mice, suggesting potential early developmental disease onset [90,91]. Besides early differentiation impairments, transcriptome analysis revealed severe downregulation of some glutamate receptor genes, like *GRIA4* (Glutamate Ionotropic Receptor AMPA Type Subunit 4) and *GRM3* (glutamate metabotropic receptor 3) [92]. Furthermore, long-term glutamate exposure led to increased cell death due to mitochondrial damage [93]. Thus, anti-glutamate drugs may offer protective effects, as the glutamate pathways seem to represent potential therapeutic targets [93].

Experiments conducted to compare patient-derived iPSC lines with CRISPR-Cas9 corrected isogenic lines, which carry a normal length of CAG repeats region within the *ATXN2* gene, revealed several pathological mechanisms, like toxic protein aggregation, disrupted RNA metabolism and mitochondrial dysfunction. This approach is pivotal to identifying therapeutic targets and testing candidate compounds under controlled, disease-relevant cellular conditions [94,95,96,97,98,99,100]. Other studies using SCA2-patient-derived iPSC models revealed that both sense (*ATXN2*) and antisense (*ATXN2-AS*) transcripts are expressed, with the expanded CUG-repeat in *ATXN2-AS* forming RNA foci that sequester *MBLN1* (Muscleblind-like 1) and cause mis-splicing of neuronal genes like *APP* (Amyloid Precursor Protein) and *GRIN1* (Glutamate Ionotropic Receptor NMDA Type 1) [101]. The *ATXN2-AS* transcript carrying the expanded CUG-repeat was also shown to be intrinsically neurotoxic, therefore potentially constituting a novel therapeutic target in SCA2 [101].

Generation of iPSCs has also been achieved from a patient affected by SCA6, an adult-onset, slowly progressive cerebellar ataxia associated with dysarthria and nystagmus linked to mutations in the gene *CACNA1A* (calcium voltage-gated channel subunit alpha 1 A). The transcript codes for two functionally unrelated proteins: the voltage-gated calcium channel (VGCC) α1A subunit, and a transcription factor, α1ACT, expressed via an internal ribosomal entry site (IRES). Interestingly, both proteins contain a polyQ tract, but only CAG expansions in α1ACT cause SCA6 [102,103]. In vitro differentiation of PCs from three SCA6 patients through a self-organizing 3D culture system exposed a previously unknown molecular mechanism, suggesting polyQ to hamper nuclear translocation of α1ACT and consequent reduction of its targets *GRN* (Granulin Precursor), *TAF1* (TATA-Box Binding Protein-Associated Factor 1) and *BTG1* (BTG Anti-Proliferation Factor 1), all important for neuronal survival. Moreover, the study showed that mutant neurons exhibited a particular vulnerability to nutrient-deprived conditions. Specifically, culturing cells in medium lacking thyroid hormone (T3)—a factor known to support PC maturation—resulted in simplified dendritic arborizations and PC degeneration [43]. Strikingly, treatment with thyrotropin-releasing hormone (TRH) to enhance T3 production, or Riluzole, a neuroprotective agent that stabilizes neural function, restored the dendritic architecture and reduced the rate of cell loss, supporting their potential use as therapeutic agents for this disease [104]. In another report, iPSCs derived from two SCA6 patients were differentiated into PCs using a different 2D-based protocol. The study confirmed the impaired transcriptional activity of α1ACT, evidenced by reduced *GRN* mRNA expression. Additionally, acute glutamate exposure resulted in enhanced excitotoxic stress, leading to the decreased viability of PCs [105].

Even though the majority of SCAs are caused by repeat expansion, including CAG repeats, several SCA types result from single nucleotide variants or small insertions/deletions in genes encoding proteins with diverse cellular functions [106]. For example, mutations in the *CACNA1G* (calcium voltage-gated channel subunit alpha 1 G) gene encoding the low-threshold voltage-dependent channel CaV3.1 have been identified as a cause of SCA42. Genetic analyses revealed a shift in channel voltage dependence toward positive potentials, significantly altering calcium channel function. Using patient-derived iPSCs, researchers successfully differentiated PCs to validate the pathogenic potential of p.Arg1715His mutation [107]. Although these iPSC-derived PCs expressed typical markers like L7 and GRID2 (glutamate ionotropic receptor delta type subunit 2) and developed characteristic dendritic structures, detailed morphometric analysis showed comparable soma diameters and dendritic field areas [107]. Considering the relevance of calcium channel dysfunction in cerebellar disease, further studies should be conducted using patient-derived iPSCs to uncover the phenotypic alterations underlying this disease.

SCA14-linked mutations in the *PRKCG* gene, which encodes the calcium-activated protein kinase C gamma (PKCγ), have also been recapitulated in iPSC-derived neurons, showing characteristic cytoplasmic PKCγ aggregation due to reduced membrane translocation in response to activation signals and insufficient degradation. This study demonstrated that the pathogenic mechanisms in SCA14 involve not only a loss of function at the membrane level but also a gain of function in the cytoplasm [108].

Neurons derived from SCA1-patient iPSCs recapitulate key disease phenotypes, including nuclear and cytoplasmic ataxin-1 protein aggregates similar to those observed in SCA1 postmortem brain tissue. In addition, SCA1-patient-derived neurons exhibited a strong bioenergetic deficit, with lower ATP production and a reduction of basal and maximal mitochondrial respiration [109]. SCA1 neurons also displayed shorter neuritic lengths, reduced dendritic branching, and delayed network activity development, clear signs of impaired neuronal maturation [109]. Transcriptomic analysis consistently identified 1050 differentially expressed genes, enriched in pathways related to synapse organization, axonal projection, and mitochondrial metabolism. Further protein–protein interaction analysis highlighted MAPK1 (Mitogen-Activated Protein Kinase 1) and RhoA (Ras homolog family member A) as the central nodes in a dysregulated signaling cluster linked to cerebellar ataxia. In the presence of mutant ataxin, the physiologically occurring ATXN1–Capicua (CIC) complex becomes hypermorphic due to the presence of polyQ expansions, resulting in altered gene repression that drives cerebellar degeneration [110]. Notably, suppressing this specific protein–protein interaction through targeted mutagenesis significantly rescues PC morphological defects and motor function without disrupting physiological CIC activity or broader cerebellar homeostasis. Moreover, transcriptomic analyses revealed a consistent downregulation of CIC target genes and dysregulation of pathways involved in synaptic function, cell adhesion and neural signaling, emphasizing the therapeutic promise for precise molecular targeting [21].

As a previously underexplored window into disease progression, researchers recently focused on identifying the earliest molecular disruptions during PC differentiation of SCA1 iPSCs. Longitudinal transcriptomic analyses showed that dysregulation of histone-related genes occurring in SCA1-patient-derived iPSCs correlated at later stages with interferon-related expression changes in PCs, including upregulation of *ISG15*, already known to be implicated in A-T’s pathology [111]. Elevation of the ISG15 protein levels, found in both cerebellar tissues of post-mortem SCA1 patients and iPS-derived PCs, apparently impairs protein degradation of ATXN1, increasing the possibility of aggregate formation [112]. Notably, ISG15 is also increased in the plasma of SCA1 patients, suggesting its potential as an early pathological biomarker. Early downregulation of the transcription factor HMGB1 (high mobility group box 1) was also previously demonstrated in SCA1’s pathology. Notably, AAV-mediated *HMGB1* delivery in SCA1 neurons partially reverted morphological deficits, supporting both the relevance and reversibility of early developmental disruptions [112,113,114]. Another study found that protein kinase A (PKA)-mediated phosphorylation of the serine 776 residue (S776) of ATXN1 in PCs contributes to the pathological accumulation of the mutant protein, suggesting that the pathological condition could be reversible by modulating PKA activity [115].

Using a completely different approach, a recent study successfully employed CRISPR-Cas9 editing methods to target and excise the expanded CAG repeat in *ATXN1* within neurons derived from SCA1-patient iPSCs, using a dual-gRNA strategy delivered via AAV [116]. Edited iPSC-derived neurons showed successful deletion of the pathogenic repeat with high specificity and no detectable off-target effects, establishing proof of concept for genome editing as a therapeutic strategy in patient-derived cells and potentially assessing phenotypic rescue post-editing [116]. Although not focused directly on a cerebellar model, another study offered important insights into SCA1-patient-derived motor neurons (MNs)—a neuronal population also implicated in the disease and linked to its lethality. SCA1 iPSC-derived MNs showed normal differentiation efficiency and neurite outgrowth but exhibited early functional and transcriptomic alterations, including impaired calcium signaling and dysregulation of genes involved in the synaptic structure and extracellular matrix interactions, pointing to early pathogenic events preceding structural degeneration [117].

Machado–Joseph disease (SCA3) is caused by a CAG repeat expansion in *ATXN3*, leading to a mutant ataxin-3 protein that tends to misfold and form toxic intracellular aggregates. iPSC-derived neurons from SCA3 patients revealed that both autophagy and molecular chaperone systems, physiologically responsible for preventing the accumulation of misfolded proteins, are actually disrupted [118,119]. In particular, SCA3-patient-derived cells display decreased expression of Beclin-1, a crucial protein in autophagy initiation [119]. Furthermore, two miRNAs (miR-340 and miR-543) that target *DNAJB1* (DnaJ heat shock protein family member B1), a gene encoding a co-chaperone protein essential for protein folding and for guiding misfolded proteins to degradation pathways, are overexpressed in SCA3-patient-derived neuroepithelial-like stem cells, leading to *DNAJB1* downregulation [118]. Interestingly, neural progenitors are relatively resistant to ATXN3 aggregation, while fully differentiated neurons show pronounced aggregation, especially after glutamate stimulation [118]. These data suggest that during differentiation, neurons, but not fibroblasts or glia, undergo profound reorganization of protein quality control systems, making them more susceptible to misfolded protein stress, such as modifications occurring in SCA3.

Gene therapy and pharmacological approaches have also been explored in iPSC models of SCA3, with targeted genetic silencing and neuroprotective interventions to modulate inflammatory responses. A preclinical study presented an adeno-associated virus (AAV)-based gene therapy approach utilizing artificial miRNAs designed to silence both mutant and wild-type *ATXN3* transcripts implicated in SCA3. The authors engineered multiple miATXN3 constructs targeting different exonic regions of the *ATXN3* mRNA, screened in vitro using luciferase reporter assays and then validated for efficacy in iPSC-derived neurons. The top-performing miATXN3 variants demonstrated robust silencing of ATXN3 at both the mRNA and protein levels without affecting non-target genes [120]. Several reports highlighted the potential of CRISPR-Cas9 technology in patient-derived iPSCs to correct pathogenic mutations and rescue cellular phenotypes associated with SCA3. In one study, Cas9-mediated genome editing was used to insert polyadenylation signals upstream of the expanded CAG repeats in the ATXN3 gene, successfully eliminating the production of mutant ataxin-3 protein. Genome-edited iPSCs were differentiated into neural progenitor cells and mature neurons, which showed a restored Golgi apparatus structure and loss of toxic ataxin-3 aggregates compared to unedited SCA3 neurons [121]. In another study, iPSCs derived from SCA3 patients were genetically corrected using CRISPR-Cas9 and differentiated into cerebellar neural stem cells and mature cerebellar neurons, including PCs (identified by the expression of L7 and CALB1), GCs (stained for markers such as ATOH1, PAX6 and ZIC2), glutamatergic neurons (positive for VGLUT1), and a small subset of GABAergic neurons (based on GABA staining) [122]. SCA3 cerebellar neurons exhibited hallmark features of the disease, such as formation of SDS-insoluble ataxin-3 aggregates and increased vulnerability to stress, which were absent in corrected cells. Notably, genetic correction also reversed disease-associated phenotypes like apoptosis and impaired proliferation, highlighting the potential of cerebellar region-specific iPSC-derived models for studying SCA3 pathogenesis and therapeutic strategies [122].

Treatments with antisense oligonucleotides (ASOs) on iPSCs derived from SCA3 patients differentiated into cortical neurons demonstrated that both general and allele-specific ASOs can effectively reduce mutant ataxin-3 expression—by up to 80%—while preserving wild-type protein levels by targeting the SNP c.987G [123]. Although this is not a cerebellar model, these iPSC-derived neurons provided a robust and disease-relevant platform to evaluate ASO efficacy, enabling rapid screening and validation. The treatments were sustained for several weeks without signs of neurotoxicity, highlighting the therapeutic promise of allele-specific gene silencing in SCA3 [123].

Besides genetic approaches, several pharmacological studies to counteract SCA3 have been performed in iPSC-derived models.

SCA3-patient-derived Purkinje progenitor cells (PPs) can be used as a rapid drug screening model [124]. SCA3 PPs, expressing neuronal and PC markers such as β-tubulin III, MAP2 and KIRREL2, exhibited the main disease features, including neurite degeneration, increased PARP1 (poly [ADP-ribose] polymerase 1) cleavage, nuclear accumulation of mutant ATXN3 fragments, and elevated calpain activity, upon excitotoxic stress with quinolinic acid (QA). Reduction of these pathological features through suppression of calpain-2 activity and promotion of calpastatin expression was obtained via treatment with N-butylidenephtalide, a compound derived from the medicinal plant *Angelica sinensis*, commonly used in traditional Chinese medicine [124,125,126]. Another study revealed that Ibuprofen, a commonly used FANS drug, has neuroprotective and therapeutic potential in models of SCA3. In particular, Ibuprofen treatments lead to lowered pro-inflammatory cytokine expression, reduced accumulation of ATXN3, and increased proliferation of neural progenitor cells. Human neurons treated with Ibuprofen also showed increased synaptophysin and neurite length [127]. In another report, SCA3-patient-derived neural progenitor cells exhibited marked proliferative defects, reduced expression of autophagy markers and a tendency to enter senescence. In this case, phenotypic reversion was obtained via activation of the STAT6 signaling pathway by treatment with interleukin-4 (IL-4), suggesting a novel therapeutic target for modulating disease progression [128]. In another study, the authors explored treatment with *Pueraria lobata* extract (NH037) and its active component Daidzein on SCA3-iPSC-derived neurons [129]. These neurons showed increased susceptibility to proteasome inhibition, with elevated oxidative stress and apoptosis compared to controls, as demonstrated by the MG132-induced cytotoxicity (MTT assay), DCFDA staining for ROS detection, TUNEL and cleaved caspase 3 assays. Daidzein treatment ameliorated these effects by enhancing ubiquitin–proteasome system activity, suggesting a potential therapeutic strategy for SCA3 [129].

Among the newly identified adult-onset hereditary ataxias, SCA27B is a pan-cerebellar syndrome with frequent oculomotor signs, found to be associated with pathological GAA-TCC repeat expansion within the first intron of the *FGF14* gene. Interestingly, the symptomatic spectrum in SCA27B resembles more the one observed in FRDA, rather than the ones described in polyQ ataxias. Functional analysis in iPSC-derived motoneurons was critical to link intronic repeat expansions with decreased mRNA and protein expression of FGF14, suggesting a gene-silencing mechanism via heterochromatinization. The study also found a correlation between repeat expansion size and earlier disease onset, even if weaker than the one reported for the other SCAs [130].

### 3.4. iPSC Modeling Revealed Developmental and DNA Damage Response Defects in Ataxia–Telangiectasia

Ataxia–telangiectasia is a rare, autosomal recessive neurodegenerative disorder caused by mutations in the *ATM* gene, which has an important role in DDR. A recent study introduced the generation of A-T patient-derived iPSC lines from olfactory mucosal biopsies. Functional studies on the iPS-derived neurons showed impaired DDR, elevated reactive oxygen species (ROS), and defective phosphorylation of key DDR targets such as CHK2 and p53 after irradiation, consistent with the known role of ATM in maintaining genomic stability. Transcriptomic analyses highlighted aberrant expressions of genes associated with cerebellar development, synaptic signaling, and oxidative stress pathways, in line with the known features of A-T pathology, like cerebellar atrophy and enhanced susceptibility to oxidative damage [131,132]. Notably, in another study where patient-derived iPSCs were differentiated into cerebellar-like neurons, several gene networks associated with cerebellar development and neuronal differentiation were disrupted, including significant downregulation of *ATOH1*, *NEUROD1*, *PTF1α* and *HOXB4*, which are critical for cell type specification and differentiation. Moreover, genes associated with synaptic function, including *SYT1*, involved in synaptic vesicle exocytosis, *SNAP25*, a core component of the SNARE complex mediating vesicle fusion, and *SLC17A6*, encoding the vesicular glutamate transporter vGLUT2, were found to be dysregulated, indicating impaired mechanisms of neurotransmitter release and, thereby, compromised synaptic transmission [133]. In agreement with these findings, another study used A-T iPSC-derived neurons to investigate how ATM deficiency impacts transcription and DDR, revealing downregulation of key genes involved in neuronal development and synaptic function, besides accumulation of DNA-Topoisomerase I complexes and impaired ATM-dependent DDR [134]. Collectively, these data on A-T iPS-derived neurons point toward a potential neurodevelopmental component of the etiopathogenesis of A-T.

### 3.5. Early Advancement in Uncovering ARSACS Pathophysiology via iPSC Modeling

Despite considerable advances in the comprehension of ataxia pathophysiology, the exact molecular and cellular mechanisms underlying less frequent cerebellar disorders, like ARSACS, are yet to be fully understood. Especially in these cases, the creation of patient-derived iPSC models offers valuable platforms to explore pathological mechanisms, identify molecular targets, and test potential therapeutic compounds.

In a recent study, iPSCs derived from three ARSACS patients and three healthy individuals were differentiated into motor neurons and PCs using both 2D and 3D culture systems. Notably, iPS-derived neurons recapitulated ARSACS features, including reduced sacsin protein expression and abnormal accumulation of neurofilaments (NF-M in particular) along neurites in both cell types. In addition, ARSACS motor neurons and PCs exhibited a consistent decrease of the mitochondrial fission factor DRP1, previously known to play an important role in ARSACS pathogenesis [135,136].

Despite these recent advances, current ARSACS iPSC-derived models remain limited, and comprehensive characterizations of disease progression, functional synaptic properties and treatment strategies are still lacking.

### 3.6. Dual Role of Microglia in Neuroinflammation and Neuroprotection in Cerebellar Ataxias

In the last two decades, the importance of microglial cells—the resident immune cells of the CNS—has gained attention in the pathogenesis of various ataxias, with a particular emphasis on their dual role as both mediators of neuroinflammation and potential neuroprotective agents [137]. The microglia represent a highly dynamic cell population that constantly surveys their environment and, under pathological conditions like ataxias, undergoes morphological and functional changes. Depending on the context, these adaptations can either exacerbate neuronal damage or promote recovery [138,139]. In a healthy brain, microglia contribute to synaptic pruning, debris clearance and maintenance of neuronal homeostasis [140]. Pro- and anti-inflammatory cytokines have critical effects on neuronal plasticity, synaptic connectivity and excitatory/inhibitory balance. In particular, TNFα and IL-6 are linked to neurodegeneration and altered neurotransmission, while TGFβ promotes neuronal survival and modulates synaptic transmission. The dysregulation of these mediators suggests that immune imbalance contributes to shared pathophysiological mechanisms across several neuropsychiatric disorders [141]. In ataxic disorders, such as SCAs, A-T and FRDA, microglial cells have been shown to adopt a pro-inflammatory phenotype characterized by the release of pro-inflammatory cytokines, including TNFα and IL-6 [142]. However, the microglial behavior is quite different in diverse forms of ataxia. For instance, in SCAs, microglial activation occurs early during development—preceding PC degeneration—and contributes to cerebellar inflammation through pro-inflammatory (M1-like) polarization, worsening disease progression. Interestingly, mutations in SCA21 are responsible for early activation of microglia and astrocytes before motor symptoms and neurodegeneration occur [143]. In FRDA, DNA damage due to frataxin deficiency can induce inflammatory microglial activation, increasing the expression of DNA repair proteins, like MUTYH (mutY DNA glycosylase) and PARP1, but also exacerbating neurological impairments. In this case, the inhibition of PARP1 seems to effectively attenuate microglial activation, reducing inflammatory damage and improving neurobehavioral outcomes [144]. Another way to target microglial inflammation was shown in an SCA6 mouse model, in which the reduction of Toll-like receptor (TLR) signaling via genetic ablation of the MyD88 adaptor protein was shown to slow disease progression [145]. Conversely, while A-T is well-known for impaired DDR and cerebellar degeneration, recent studies revealed that loss of ATM function leads to the presence of cytosolic DNA inside cells, which triggers an activated pro-inflammatory microglial phenotype, ultimately exacerbating PC loss [146]. A-T iPSC-derived microglia actually exhibited increased production of inflammatory cytokines, dysregulation of NF-kB signaling, oxidative stress and altered phagocytic activity, collectively capable of inducing dendritic retraction and apoptosis in wild-type PCs in co-culture experiments, supporting a central role of microglia in neurotoxicity. Notably, functional rescue was achieved via pharmacological inhibition of NF-kB and by reducing ROS using N-acetylcysteine, both of which restored microglial homeostasis and protected PCs in vitro and in vivo [146]. Using a more generic approach, anti-inflammatory therapy with betamethasone was reported to improve neurological symptoms in A-T patients, and it was shown to reduce microglial activation, inflammation and neuronal loss in ATM-deficient rats [147,148]. Importantly, while chronic microglial activation can contribute to neurodegeneration, this cell type also plays a crucial role in synaptic remodeling and tissue repair, particularly during early disease stages or in response to injury, making the microglia key players in the progression and modulation of cerebellar ataxias [149,150].

### 3.7. Expanding the Frontiers of Ataxia Research

Beyond neuronal loss and neuroinflammation, recent evidence highlights the involvement of the cerebellum in modulating broader aspects of nervous system plasticity and recovery. In particular, a large-scale clinical database analysis of over 9 million patients showed that individuals with ataxias failed to recover from common peripheral nerve injury (PNI) (e.g., carpal tunnel syndrome), unlike non-ataxia controls. The cerebellar glutamatergic system appears to be involved in the recovery process, as ionotropic glutamate receptor GRIA1 is normally upregulated in PNI. Conversely, expression of *GRIA1* is not modified in the cerebellar tissue of A-T patients and iPSC-derived PCs from SCA6 patients, suggesting that impaired glutamatergic signaling in the DCN underlies the core of this pathological mechanism [151].

Considering another disorder related to ataxias, researchers are building a clinical-trial-ready patient cohort for multiple system atrophy (MSA), a rapidly progressive and fatal neurodegenerative disorder with no effective treatments [152]. Even if MSA is not a hereditary ataxia like SCAs, it is grouped within sporadic adult-onset ataxias. In particular, the cerebellar subtype of MSA (MSA-C) shares the main symptoms with cerebellar ataxias, including gait disturbance, dysarthria and cerebellar degeneration [153]. Also, in this case, patient-derived iPSC lines demonstrated high reliability and predictive power, as neurons differentiated from MSA-C patients carrying mutations in the *COQ2* gene, an enzyme involved in CoQ10 biosynthesis, showed reduced levels of CoQ10 and vitamin E, with consequent impairments in mitochondrial respiration and elevated oxidative stress. Interestingly, correction of the *COQ2* mutation via CRISPR/Cas9 partially rescued mitochondrial function and reduced oxidative stress, supporting a direct role of this mutation in MSA pathophysiology [154].

In another case regarding ataxia-related diseases, researchers generated patient-derived iPSCs harboring mutations in the *CEP290* gene, frequently altered in Joubert syndrome (JS). JS is a ciliopathy-associated neurodevelopmental disorder that often presents cerebellar hypoplasia, ataxia and cognitive impairment [155]. Despite *CEP290*-mutant iPSCs being capable of forming NPCs, neuronal maturation is significantly impaired, as seen by the reduction of MAP2, DCX, and TUBB3 markers, abnormal cellular morphology as reduced neurite length and branching, and altered electrophysiological properties. Transcriptomic profiling of JS-patient-derived neurons confirmed the dysregulation of signaling pathways, such as WNT, SHH and TGF-β, crucial to cerebellar development, reflecting the cerebellar vermis hypoplasia and impaired motor coordination frequently observed in JS patients [156,157].

Overall, iPSC technology has significantly advanced the modeling of cerebellar ataxias by enabling patient-specific and physiologically relevant studies of early disease mechanisms and therapeutic targets. To fully capture the genetic and clinical diversity of ataxias, future efforts should focus on generating a broader collection of iPSC-based models and promoting biomarker discovery. An overview of key findings covered in the text, along with relative disease and iPSC models, is presented in Table 1.

## 4. Discussion

iPSC-derived models have emerged as promising platforms for studying cerebellar ataxias, offering patient-specific systems that better recapitulate human developmental and pathological features than traditional animal models [158]. Protocols have evolved from basic patterning to advanced differentiation methods that yield long-term matured cerebellar organoids, recapitulating fetal cerebellar cellular diversity [41,43]. These models have shown success in generating RL- and VZ-derived lineages and even enabled disease modeling [42]. Moreover, 3D Matrigel-based cultures and glial co-culture have been essential in supporting dendritic arborization, axonal projection and PC maturation [76]. Despite these achievements, significant challenges remain. Generation of fully mature and functional cerebellar neurons in vitro is still limited by incomplete knowledge of human-specific developmental cues and the complexity of cerebellar cytoarchitecture, together with the known limitations associated with commonly used iPSC-based models. In particular, chromosomal aberrations and other mutations can occur during the reprogramming process [159,160]. Moreover, besides the reported possibility that iPS-derived cells could remain developmentally immature, resembling fetal rather than adult stages, the vast majority of 2D and 3D models lack the full cellular diversity, vascularization and broad network connectivity of the human brain [161]. The variability between lines, high passaging, culture media, and differentiation protocols also affects reproducibility, while long-term culture and maturation remain technically challenging [162]. However, iPSCs remain a highly promising model system, considering their unique ability to combine patient-specific genetic background with highest differentiation capacity. Future development of novel protocols for generating even more complex iPSC-based systems will potentially unleash the full recapitulation of integrated human neurological disorder mechanisms, providing the foundation for the discovery of innovative therapeutic strategies [163,164].

The usefulness of iPSC-derived models in ataxias has surged in the last decade. For instance, patient-derived iPSCs from A-T and SCA cases have revealed critical PC vulnerabilities, including disrupted gene regulatory networks, increased sensitivity to stressors like thyroid hormone depletion and key pathogenetic mechanisms, highlighting the value of iPSCs not only for disease modeling but also for uncovering new therapeutic targets [21,104,115,133]. Importantly, recent findings suggest that neurodevelopmental alterations, including chromatin remodeling and immune priming, precede the onset of classic neurodegenerative features in SCA1 [112]. Furthermore, iPSC models of JS have elucidated the role of primary cilia in neural differentiation and synaptic maturation, establishing a mechanistic link between *CEP290* mutations and neurodevelopmental deficits [156].

Similarly, even if *ATM* is crucial for responding to DNA damage, it is not required for the expression of activity-induced immediate early genes [134]. Collectively, these new pieces of evidence point to the emerging view that abnormalities in cerebellar development could not be mere secondary consequences but may serve as upstream drivers of adult-onset cerebellar degeneration, highlighting the urgency of developing therapeutic interventions targeting the earliest stages of disease progression. From another point of view, the successful modeling of cerebellar pathology in iPSC-derived neurons has enabled researchers to replicate key disease mechanisms and provided a valuable system for testing therapies aimed at restoring protein localization or enhancing protein degradation pathways, such as cytoplasmic PKCγ aggregation in SCA14 mutant cells [108]. Similarly, FRDA patients’ iPSC-based models have facilitated the identification of promising therapeutic strategies, such as GLP-1R agonists, which enhance frataxin expression and improve mitochondrial function [84]. Additionally, therapeutic repurposing of existing drugs holds promise in iPSC research: Ibuprofen has demonstrated neuroprotective effects in SCA3 models by reducing neuroinflammation and supporting neural regeneration, while approaches aimed at modulating autophagy and restoring calcium homeostasis are under investigation for SCA2 [88,127].

Besides pharmacological approaches, gene therapy represents a rapidly advancing field, with knockdown strategies for mutant transcripts showing significant promise. In particular, the positive effects observed for antisense oligonucleotide therapies targeting *ATXN2* expression, together with the efficacy demonstrated by AAV5-miATXN3 vectors in SCA3 cellular models, could provide a foundation for the clinical translation of RNAi-based therapies, potentially extendable to polyglutamine disorders and other neurological diseases due to protein aggregation [88,120]. Finally, therapeutic modulation of microglia is emerging as an important approach due to their dual role in both neuroprotection and neurodegeneration [150]. Current strategies focus on modulating microglial activation through anti-inflammatory agents (e.g., Ibuprofen, Minocycline), polarization modulators and mitochondrial support [149]. Studies in A-T models showed that controlling microglial activation can attenuate neuroinflammation and prevent PC degeneration [146], highlighting the microglia as promising therapeutic targets across multiple ataxias. However, further studies are needed to better understand the timing, localization and heterogeneity of microglial responses, with the ultimate goal of enhancing their beneficial properties while mitigating their damaging effects. Recently, it has been discovered that in low inflammatory conditions, genetic variants of the serotonin receptor 5-HTR2A strongly influence psychiatric disorder phenotypes, while the genetic contributions are weaker under pro-inflammatory conditions, suggesting that immune-mediated epigenetic regulation can affect receptor signaling participating in the clinical variability of mental illness [165]. Furthermore, glial cells release soluble adhesion molecules that modulate blood–brain barrier permeability and guide immune cell entry, thereby establishing a link between systemic immune activation and neuroinflammatory processes, capable of influencing neurotransmitter signaling with downstream effects on neuronal functions [166]. These immune-mediated mechanisms should also be investigated within cerebellar processes to investigate their potential contribution to the pathophysiology of ataxias.

Regarding ultra-rare ataxias such as ARSACS, the development of patient-derived iPSC models offers a promising strategy to dissect disease mechanisms at the molecular and cellular levels. These models, indeed, not only enable genotype–phenotype correlation but also support precision medicine approaches [33,153].

Overall, there is a critical need to expand the repertoire of iPSC-based models carrying diverse pathological genetic variations, as they represent an indispensable tool for advancing the mechanistic understanding of early molecular disruptions and potential therapeutic targets that traditional models cannot fully recapitulate. By preserving patient-specific genetic backgrounds, iPS-derived neurons may serve as a powerful platform for high-throughput drug screening and therapeutic target identification, ultimately refining treatment strategies more precisely. For all these reasons, building comprehensive, well-characterized iPSC biobanks integrated with clinical phenotyping is essential for translating these advances into effective treatments for currently intractable cerebellar ataxias.

## Figures and Tables

**Table 1:** Summary of the cerebellar ataxias discussed in the text and the associated iPS models. The main results are listed, together with the proposed therapeutical approaches.

Disease	Gene	Models	Main Results	References
Friedrich’s Ataxia	*FXN*	FRDA patients’ iPSC-derived neurons	Exenatide treatments enhance frataxin expression, improve oxidative stress and alleviate mitochondrial dysfunction. Frataxin deficiency-induced DNA damage leads to microglial activation.	[78,84,144]
Spinocerebellar Ataxia 1 (SCA1)	*ATXN1* *ISG15*	SCA1 patients’ iPSC-derived neurons and Purkinje cells	Expanded ATXN1 protein forms a hyperactive complex with CIC, leading to altered gene expression that contributes to cerebellar degeneration. ISG15 could be an early SCA1 biomarker. PKA-S776 signaling could be a promising therapeutic target, as PKA seems to be an upstream regulator of *ATXN1*.	[21,112,115]
Spinocerebellar Ataxia 2 (SCA2)	*ATXN2*	SCA2 patients’ iPSC-derived neurons	Relevant screening platforms for evaluating new therapeutic targets. Disruptions to calcium channel function lead to excitotoxicity and impaired LTD. SCA2 NSCs suggest early developmental alterations. Transcriptome analysis reveals downregulation of glutamate receptor genes.	[88,90,93,100]
Spinocerebellar Ataxia 3 (Machado–Joseph Ataxia or SCA3)	*ATXN3*	SCA3 patients’ iPSC-derived neurons, cerebellar cells and progenitors	Disrupted autophagy and chaperone pathways, with reduced Beclin-1 and DNAJB1 expressions. Mutant ATXN3 aggregation is more pronounced in mature neurons than in progenitors. AAV-based gene therapy using engineered miRNAs silences both mutant and wild-type *ATXN3*. N-butylidenephtalide reverses excitotoxic stress. Ibuprofen treatments reduce pro-inflammatory cytokine levels and decrease ATXN3 accumulation. IL-4 activates STAT6 signaling, thereby restoring proliferation and enhancing autophagy-related gene expression. Daidzein alleviates oxidative stress and apoptosis.	[118,120,124,127,128,129]
Spinocerebellar Ataxia 6 (SCA6)	*CACNA1A*	SCA6 patients’ iPSC-derived Purkinje cells	PolyQ-expanded α1ACT protein fails to translocate to the nucleus, reducing expression of neuroprotective genes. T3 decrement leads to simplified dendritic arborizations and cell death, rescued by TRH and Riluzole. Increased susceptibility to glutamate-induced excitotoxicity. Genetic ablation of *MyD88* reduces microglial activation and slows disease progression.	[77,102,103,104,105,145]
Spinocerebellar Ataxia 42 (SCA42)	*CACNA1G*	SCA patients’ iPSC-derived Purkinje cells	Channel Cav3.1 exhibits a shift in the voltage sensitivity toward more depolarized potentials, disrupting calcium signaling.	[107]
Spinocerebellar Ataxia 14 (SCA14)	*PRKCG*	SCA14 patients’ iPSC-derived neurons	PKCγ fails to translocate properly to the membrane, leading to cytoplasmic aggregation and impaired degradation, resulting in membrane-level loss-of-function and cytoplasmic gain-of-function effects.	[108]
Spinocerebellar Ataxia 27B (SCA27B)	*FGF14*	SCA27B-patient-derived iPSCs	A GAA-TCC intronic repeat expansion was identified, with larger expansions linked to reduced FGF14 expression, likely due to heterochromatin-mediated silencing.	[130]
Ataxia–Telangiectasia (A-T)	*ATM*	A-T patients’ iPSC-derived neurons	Olfactory mucosal biopsy for generating iPSC lines. Derived neurons analyses show impaired cerebellar development, synaptic signaling and oxidative stress pathways. Loss of ATM function leads to a hyperactive microglial phenotype, promoting neurodegeneration, which seems to be reduced by betamethasone treatment.	[131,132,133,146,148]
Autosomal Recessive Spastic Ataxia of Charlevoix–Saguenay	*SACS*	ARSACS patients’ iPSC-derived Purkinje cells and motor neurons	Derived neuron types revealed reduced sacsin protein expression and abnormal accumulation of NFM along neurites.	[136]
Peripheral Nerve Injury (PNI)	*GRIA1*	SCA6 patients’ iPSCs-derived PCs and A-T patients’ cerebellar tissue	Reduced *GRIA1* expression in ataxia patients disrupts the excitatory-inhibitory balance in DCN, impairing motor recovery after PNI.	[151]
Multiple System Atrophy (MSA)		MSA patients’ iPSC-derived neurons	Derived neurons show α-synuclein accumulation, glial cytoplasmic inclusions and alterations in neuroinflammatory responses	[153]
Joubert Syndrome (JS)	*CEP290*	CEP290-mutant iPSC-derived NPCs	Mutant cells show reduced expression of neural maturation markers, fail to form mature neurons and neuronal networks.	[156]
